# DiTing: A Pipeline to Infer and Compare Biogeochemical Pathways From Metagenomic and Metatranscriptomic Data

**DOI:** 10.3389/fmicb.2021.698286

**Published:** 2021-08-02

**Authors:** Chun-Xu Xue, Heyu Lin, Xiao-Yu Zhu, Jiwen Liu, Yunhui Zhang, Gary Rowley, Jonathan D. Todd, Meng Li, Xiao-Hua Zhang

**Affiliations:** ^1^College of Marine Life Sciences, and Frontiers Science Center for Deep Ocean Multispheres and Earth System, Ocean University of China, Qingdao, China; ^2^Laboratory for Marine Ecology and Environmental Science, Qingdao National Laboratory for Marine Science and Technology, Qingdao, China; ^3^School of Earth Sciences, University of Melbourne, Parkville, VIC, Australia; ^4^Institute of Evolution & Marine Biodiversity, Ocean University of China, Qingdao, China; ^5^School of Biological Sciences, University of East Anglia, Norwich Research Park, Norwich, United Kingdom; ^6^Shenzhen Key Laboratory of Marine Microbiome Engineering, Institute for Advanced Study, Shenzhen University, Shenzhen, China

**Keywords:** biogeochemical cycle, metagenomics, pipleline, software, DiTing, metatranscriptomics

## Abstract

Metagenomics and metatranscriptomics are powerful methods to uncover key micro-organisms and processes driving biogeochemical cycling in natural ecosystems. Databases dedicated to depicting biogeochemical pathways (for example, metabolism of dimethylsulfoniopropionate (DMSP), which is an abundant organosulfur compound) from metagenomic/metatranscriptomic data are rarely seen. Additionally, a recognized normalization model to estimate the relative abundance and environmental importance of pathways from metagenomic and metatranscriptomic data has not been organized to date. These limitations impact the ability to accurately relate key microbial-driven biogeochemical processes to differences in environmental conditions. Thus, an easy-to-use, specialized tool that infers and visually compares the potential for biogeochemical processes, including DMSP cycling, is urgently required. To solve these issues, we developed DiTing, a tool wrapper to infer and compare biogeochemical pathways among a set of given metagenomic or metatranscriptomic reads in one step, based on the Kyoto Encyclopedia of Genes and Genomes (KEGG) and a manually created DMSP cycling gene database. Accurate and specific formulae for over 100 pathways were developed to calculate their relative abundance. Output reports detail the relative abundance of biogeochemical pathways in both text and graphical format. DiTing was applied to simulated metagenomic data and resulted in consistent genetic features of simulated benchmark genomic data. Subsequently, when applied to natural metagenomic and metatranscriptomic data from hydrothermal vents and the *Tara* Ocean project, the functional profiles predicted by DiTing were correlated with environmental condition changes. DiTing can now be confidently applied to wider metagenomic and metatranscriptomic datasets, and it is available at https://github.com/xuechunxu/DiTing.

## Introduction

Biogeochemical cycles mainly refer to the movement of chemical substances (e.g., carbon, nitrogen, and sulfur) between the biotic and the abiotic compartments, which impact climate change and human health (Rousk and Bengtson, 2014; Abatenh, 2018). Microbial communities play integral and unique roles in mediating global biogeochemical cycles. Applications of sequencing techniques, such as amplicon sequencing (Bokulich et al., 2013), whole-genome sequencing (Jones and Good, 2016; Xue et al., 2020b), genome-resolved metagenomics (Parks et al., 2017), and shotgun metagenomic sequencing (Sharpton, 2014; Xue et al., 2020a), are used widely to characterize the genetic potential of microbial communities. Metagenomics is an important tool to unravel the diversity, function and ecology of complex microbial ecosystems, via quantification of the genetic potential for various biogeochemical pathways within microbial communities (Riesenfeld et al., 2004; Pinnell and Turner, 2019). Moreover, metatranscriptomic data present more accurate scenarios of processes occurring within ecosystems because these methodologies move past genetic potential and report on the transcription of biogeochemical pathway genes (Aguiar-Pulido et al., 2016; Shakya et al., 2019). Previous studies have predicted community functions according to gene annotation against several established databases, e.g., Kyoto Encyclopedia of Genes and Genomes (KEGG) (Ogata and Goto, 2000), COG (Tatusov et al., 2000), MetaCyc (Caspi et al., 2006), Pfam (Finn et al., 2014), TIGRfam (Selengut et al., 2007), SEED (Ross et al., 2014), and eggNOG (Huertacepas et al., 2016). However, these functional annotations are not dedicated to biogeochemical cycling and lack comprehensive lists of annotated genes for important cycles. Another tool, Functional Ontology Assignments for Metagenomes (FOAM), although including biogeochemical cycling genes, does not permit visualization to facilitate interpreting functional profiles, and it annotates all protein sequences with a universal threshold value, which may lead to prediction biases (Prestat et al., 2014). Some tools can be used in the analysis of genome, metagenome or metatranscriptome, e.g., METABOLIC (Zhou et al., 2020), iPATH (Darzi et al., 2018), gapseq (Zimmermann et al., 2021), MEGAN (Huson et al., 2007), and SAMSA2 (Westreich et al., 2018). The METABOLIC (Zhou et al., 2020) toolkit can assess microbial ecology and biogeochemistry based on evaluating the completeness of pathways in genomes or/and metagenome-assembled genomes, but is not directly based on calculating the relative abundance of pathways. iPath (Darzi et al., 2018) and gapseq (Zimmermann et al., 2021) are applications for the visualization and analysis of metabolic pathways in a cellular genome or a set of gene sequences, but not metagenomes. These two applications do not specialize in the biogeochemical cycle and cannot calculate the relative abundance of pathways. MEGAN (Huson et al., 2007) is a program to analyze the taxonomical content of metagenomes, but cannot access functional profiles. SAMSA2 is a metatranscriptome analysis pipeline that can determine differing features between individual metatranscriptomes (Westreich et al., 2018), but it does not focus on the biogeochemical cycle-related genes and pathways, and cannot deal with metagenome. Furthermore, some biogeochemical pathways, e.g., the cycling of dimethylsulfoniopropionate (DMSP), which is a key marine osmolyte, nutrient and signaling molecule with important roles in sulfur cycling (Curson et al., 2011; Zhang et al., 2019), lack accurate and reviewed databases for annotating the key metabolic genes. Although a sulfur cycle database SCycDB (Yu et al., 2021) published very recently includes most of the marker genes of the DMSP cycle, some key genes are not included, such as eukaryotic-type methyltransferase DSYB and acryloyl-CoA hydratase AcuH. These limitations force researchers to undertake often tricky and time-consuming gathering of gene sequences from primary research and collate them into local databases (Llorens-Marès et al., 2015; Dombrowski et al., 2018; Zhang et al., 2018; Acinas et al., 2019). Also, this may lead to challenges for downstream interpretation, organization, and visualization.

Additionally, to infer the relative abundance of pathways for metagenomic and metatranscriptomic data, there is not any prepared normalization method for pathways involving multiple genes. In some studies, the relative abundance of every gene in a biogeochemical pathway was added together (Petter et al., 2013; Smedile et al., 2013; Ganesh et al., 2014). The cumulative relative abundance is not suitable for comparing different pathways within a sample, thus we consider a method that can calculate the average relative abundance of all genes in a pathway. For example, thiosulfate disproportionation (thiosulfate→sulfide and sulfite) is catalyzed by thiosulfate reductase, which is encoded by three genes (*phs-A*, *B*, and *C*, hereafter collectively referred to *phsABC*) (Heinzinger et al., 1995). Thus, the relative abundance of the thiosulfate disproportionation pathway should be the mean relative abundance of *phsABC* instead of the sum of *phsABC* relative abundance when compared to other sulfur-related pathways within a sample. This normalization mode was applied in some recent studies (Llorens-Marès et al., 2015; Graham et al., 2018). However, there is a lack of any simple tools to achieve this normalization. Also, handy methods for high throughput comparison and visualization of samples are rarely seen. Therefore, new automated tools to identify, quantify, and compare the abundance and/or transcription of genes and pathways for biogeochemical cycles, including the DMSP cycle, are needed.

Here we developed the software DiTing, which is a pipeline to infer and compare biogeochemical pathways in metagenomic and metatranscriptomic data. DiTing is named after a Chinese mythical creature who knows everything when he put his ears on the Earth’s surface. Similarly, scientists may gain robust knowledge on microbial-driven biogeochemical cycles from environmental ‘omic data after analysis with DiTing. DiTing annotates protein sequences based on the KEGG database (Ogata and Goto, 2000) for most microbial-mediated biogeochemical cycles supplemented with a supervised database developed specifically for DMSP cycling. The relative abundance of each functional gene was calculated followed by the relative abundance of each pathway, which is calculated according to a customized formula. The output results consist of summary tables conveniently presenting over 100 biogeochemically relevant pathways and corresponding genes with their relative abundances in individual metagenomic/metatranscriptomic samples. This is alongside graphical outputs consisting of heatmaps and multiple sketch plots for easier visualization and comparison. We applied DiTing to simulated benchmark metagenomic data and natural real metagenomic and metatranscriptomic data, which demonstrated the accuracy of this tool and its potential application in the environmental microbiome.

## Materials and Methods

### The Main Procedure of DiTing

#### Assembly

**FIGURE 1:**
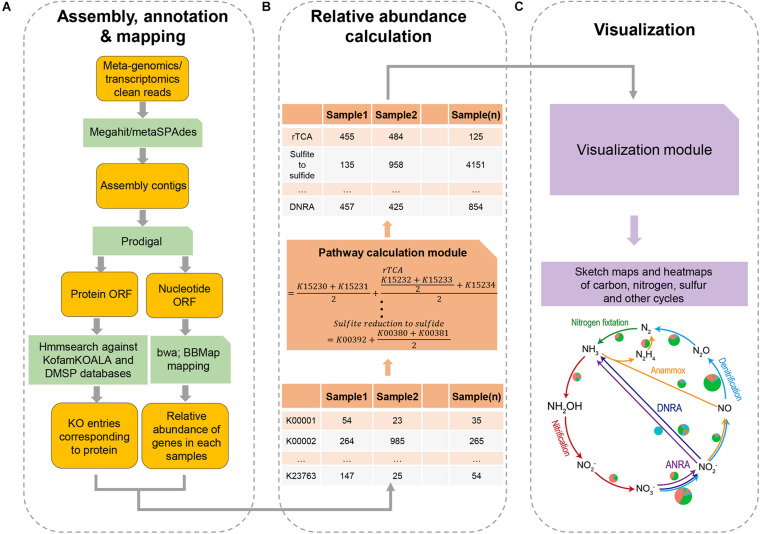
A flowchart of the major steps involved in running DiTing. First **(A)**, clean reads of metagenomes or/metatranscriptomes are assembled, annotated, and mapped. Second **(B)**, a table for relative abundances of KO number in KEGG among samples is constructed and relative abundances of biogeochemical pathways are estimated according to unbiased specific formulas. Third **(C)**, heatmap and sketch plots are drawn to aid visualization.

DiTing was written in Python 3 and runs on Linux/Unix platforms. The pre-requisites required for running the software are described on the DiTing GitHub page^1^. DiTing can be installed via Conda^2^. The input source is a set of metagenomic and/or metatranscriptomic clean reads where low-quality reads, primer, and adaptor sequences have been trimmed beforehand (Figure 1). The input datasets are then assembled by Megahit v1.1.2 (Li et al., 2016) or metaSPAdes v3.12.0 (Nurk et al., 2017) with the assembler’s default parameters according to users’ specification. Compared to Megahit, MetaSPAdes performs better in recovering long contigs. It has a higher assembly quality index and is the recommended assembler for high-complex metagenomes (Forouzan et al., 2018; Pasolli et al., 2019). However, Megahit has a low error rate, is highly memory-efficient, and is ideal for large datasets (Forouzan et al., 2018). Optionally, users can also assemble reads by themselves before running DiTing. DiTing supports assembled contigs and clean reads together as input.

#### Gene Prediction and Quantification

Genes are predicted and translated from the assembled contigs by Prodigal v2.6.3 with the “-p meta” option (Hyatt et al., 2010). To determine the relative abundance of each gene, the input metagenomic reads are mapped against predicted genes (nucleotides) by BWA-MEM (Li, 2013) (bwa v0.7.15, default settings) to generate sequence alignment map (SAM) files. Unsorted SAM files are used as input for pileup.sh (bbmap v38.22) (Bushnell, 2014, default parameters) to calculate the average coverage of each gene or transcript. The TPM methodology is used to indicate the relative abundance of a gene by the following formula.

$$T\,P\,M_{i}=\frac{b_{i}}{\sum_{j}b_{j}}\cdot 10^{6}=\frac{\frac{X_{i}}{L_{i}}}{\sum_{j}\frac{X_{j}}{L_{j}}}\cdot 10^{6}$$

where *TPM*_*i*_ is the relative abundance of gene *i*, *b*_*i*_ is the copy number of gene *i*, *L*_*i*_ is the length of gene *i*, *X*_*i*_ is the number of times that gene *i* is detected in a sample (i.e., the number of reads in alignment), and *j* is the number of genes in a sample.

#### Gene Annotation

The translated protein sequences are queried against KOfam database [HMM database of KEGG Orthologs (KOs)] (Aramaki et al., 2019) using hmmsearch implemented within HMMER (Finn et al., 2011) (parameter: hmmsearch -T <*threshold*> –tblout <output> <hmm database><input protein sequence> when the score type is “full”; hmmsearch –domT <*threshold*> –domtblout < output> <hmm database><input protein sequence> when the score type is “domain”). This employs methods for detecting remote homologs sensitively and efficiently. KOfam suggested values^3^ are used as the cutoff threshold values for hmmsearch, in which each KO entry has its unique cutoff threshold values (Aramaki et al., 2019). KofamKOALA assigns KOs numbers to protein sequences with the accuracy being comparable to the best existing KO assignment tools (Aramaki et al., 2019). For genes assigned into multiple KOs numbers, all the corresponding functions are associated with the genes. To specifically probe DMSP catabolism, 20 verified gene sequences (DMSP lyase genes *dddD*, *dddK*, *dddL*, *dddP*, *dddQ*, *dddY*, *dddW*, *Alma1*; DMSP synthesis genes *dsyB*, *DSYB*, *mmtN*; DMSP demethylation pathway genes *dmdA*, *dmdB*, *dmdC*, *dmdD*; acryloyl-CoA hydratase *acuH*, methanethiol *S*-methylase *mddA*, dimethyl sulfide (DMS) monooxygenase *dmoA*, methanethiol oxidase *MTO*, and DMSO reductase *dorA*) were collected manually to create the profile HMM using HMMER 3.3.1 (Eddy, 2011). A table with the relative abundance and annotation of genes was used to estimate the relative abundance of approximately one hundred biogeochemical pathways in each sample.

#### Normalization

The formula for each pathway is specifically designed to estimate the relative abundance of the pathway according to the definitions^4^ :

$$A_{i}\,=\frac{a_{1\,\_\,1}+a_{1\,\_\,2}+a_{1\,\_\,n}}{n}+\frac{a_{2\,\_\,1}+a_{2\,\_\,2}+\ldots +a_{2\,\_\,n}}{n}+\ldots +\frac{a_{m\,\_\,1}+a_{m\,\_\,2}+\ldots +a_{m\,\_\,n}}{n}$$

where *A*_*i*_ is the relative abundance of the *i* pathway, and *a*_*m_n*_ is the relative abundance of protein *m_n* in each sample. *m* is one of the optional routes for accomplishing the *i* pathway, and *n* is the number of proteins in the optional route *m*. For example, assimilatory sulfite reduction (ASR) that converts sulfite to sulfide has two known possible pathways: (1) Sir protein (K00392) mediated pathway (Gisselmann et al., 1993; Bork et al., 1998), and (2) CysJI protein (K00380 + K00381) mediated pathway (Ostrowski et al.,1989a,b; Zeghouf et al., 2000). Thus, the relative abundance of ASR pathway is estimated by the following formula:

$$A_{A\,S\,R}\,=a_{K\,00392}+\frac{a_{K\,00380}+a_{K\,00381}}{2}$$

where *A*_*ASR*_ is the relative abundance of the ASR pathway, *a*_*KO*_ is the relative abundance of KO in each sample. Dissimilatory nitrite reduction (DNRA), which converts nitrite to ammonia, may occur via two different enzymatic reactions: (1) *NirBD* proteins (K00362 + K00363) to convert nitrite to ammonia, or (2) *NrfAH* protein (K03385 + K15876) to convert nitrite to ammonia. Thus, the relative abundance of DNRA to ammonia is estimated by the following formula:

$$A_{D\,N\,R\,A}=\frac{a_{K\,00362}+a_{K\,00363}}{2}+\frac{a_{K\,03385}+a_{K\,15876}}{2}$$

where *A*_*DNRA*_ is the relative abundance of DNRA pathway, *a*_*KO*_ is the relative abundance of KO in each sample. For other pathways, a customized formula for each pathway was utilized (see Supplementary Table 1).

DiTing produces a table in the specified output directory. This table contains approximately 100 biogeochemical pathways and their relative abundance in each input sample. Another table of the relative abundances of corresponding KO/genes within these pathways in each sample is also generated. For improved visualization, heatmaps and sketch plots for comparing the relative abundances of biogeochemical pathways in different samples are drawn finally. Output also contains some important intermediate data, such as assembled contig, gene sequence and mapping file.

### Construction of the Organosulfur Compound Database

Dimethylsulfoniopropionate is a marine organosulfur compound with important roles in the global sulfur cycle and may affect climate (Zhang et al., 2019). Yet, genes involved in the cycling of this compound are rarely seen in currently available databases. Profile HMM were manually generated for eight pathways related to the cycling of DMSP (Song et al., 2020; Yu et al., 2021), including DMSP biosynthesis (methionine→DMSP), DMSP demethylation (DMSP→MMPA), DMSP demethylation (MMPA→MeSH), DMSP cleavage (DMSP→DMS), DMS oxidation (DMS→MeSH), DMS oxidation (DMS→DMSO), DMSO reduction (DMSO→DMS), MddA pathway (MeSH→DMS), MeSH oxidation (MeSH→Formaldehyde). Twenty verified gene sequences encoding key enzymes of these pathways were used to create the profile HMM (Song et al., 2020). Each separate cut-off *E*-value was confirmed by blasting between functionally verified protein sequences. We applied this *E*-value to several metagenomic samples to retrieve homologs. All retrieved homolog sequences were aligned to the verified protein sequences and then a maximum likelihood phylogenetic tree was constructed to further ensure the accuracy of the *E*-value. The custom HMM databases are available for download and can be used in other pipelines as well.

#### DMSP Biosynthesis (Methionine→DMSP)

Three gene families participating in DMSP biosynthesis from methionine (Met), including DSYB, DsyB, and MmtN are included in DiTing. DSYB and DsyB are methylthiohydroxbutryrate *S*-methyltransferase enzymes found in marine eukaryotes and prokaryotes, respectively (Curson et al., 2017, 2018). The MmtN Met *S*-methyltransferase is found in some Gram-positive bacteria, alpha- and gamma-proteobacteria (Liao and Seebeck, 2019; Williams et al., 2019). The cut-off *E*-values of DSYB, DsyB, and MmtN are 1 × 10^–30^, 1 × 10^–67^, and 1 × 10^–98^, respectively.

#### DMSP Demethylation (DMSP→MMPA)

The first step of DMSP demethylation pathway that results in the production of methylmercaptopropionate (MMPA) is initiated by the DmdA enzyme (Reisch et al., 2011a). The cut-off *E*-value of the DmdA is 1 × 10^–130^.

#### DMSP Demethylation (MMPA→MeSH)

Further degradation of MMPA generating gaseous methanethiol (MeSH) catalyzed by the Dmd- B, C, and D (hereafter collectively referred to DmdBCD) or AcuH enzymes (Reisch et al., 2011b; Shao et al., 2019). The cut-off *E*-values of DmdB, DmdC, DmdD, and AcuH are 1 × 10^–75^, 1 × 10^–100^, 1 × 10^–30^, and 1 × 10^–56^, respectively.

#### DMSP Cleavage (DMSP→DMS)

Eight distinct DMSP lyase enzymes (DddD, DddK, DddL, DddP, DddQ, DddW, DddY and Alma1) can cleave DMSP to generate DMS (Curson et al., 2011; Alcolombri et al., 2015; Johnston et al., 2016; Sun et al., 2016). The cut-off *E*-values of DddD, DddK, DddL, DddP, DddQ, DddW, DddY, and Alma1 are 1 × 10^–97^, 1 × 10^–35^, 1 × 10^–33^, 1 × 10^–83^, 1 × 10^–20^, 1 × 10^–49^, 1 × 10^–64^, and 1 × 10^–26^, respectively.

#### DMS Oxidation (DMS→MeSH)

Dimethylsulfoniopropionate can be oxidized to generate MeSH via the DMS monooxygenase enzyme DmoA (Boden et al., 2011). The cut-off *E*-value of the DmoA is 1 × 10^–34^.

#### DMS Oxidation (DMS→DMSO)

Dimethylsulfoniopropionate can be oxidized to generate dimethyl sulfoxide (DMSO) by the DMS dehydrogenase complex (DdhABC) (McDevitt et al., 2002) or trimethylamine monooxygenase (Tmm) (Lidbury et al., 2016). The cut-off *E*-values of both DdhABC, DdhB, and Tmm are 1 × 10^–30^.

#### MddA Pathway (MeSH→DMS)

MeSH can be *S*-methylated to generate DMS by the MddA enzyme (Carrión et al., 2017). The cut-off *E*-value of MddA is 1 × 10^–30^.

#### MeSH Oxidation (MeSH→Formaldehyde)

MeSH can also be modified through another pathway catalyzed by the MeSH oxidase MTO (Eyice et al., 2018). The cut-off *E*-value of MTO is 1 × 10^–20^.

The sugar 6-deoxy-6-sulfoglucose (sulfoquinovose, SQ), which is produced by plants, algae, and cyanobacteria, is an important component of carbon and sulfur cycles (Frommeyer et al., 2020). The microbial community can completely degrade SQ into inorganic sulfate or hydrogen sulfide through three pathways, i.e., sulfo-Embden-Meyerhof-Parnas (sulfo-EMP) (Denger et al., 2014), sulfo-Entner-Doudoroff (sulfo-ED) (Felux et al., 2015), and 6-deoxy-6-sulfofructose-transaldolase (SFT) pathways (Frommeyer et al., 2020).

#### Sulfo-EMP Pathway

Sulfoquinovose is converted to 6-deoxy-6-sulfofructose (SF) through an aldose/ketose isomerase YihS. The SF is phosphorylated to 6-deoxy6-sulfofructosephosphate (SFP) by an ATP-dependent SF kinase YihV. The SFP is then cleaved into 3-sulfolactaldehyde (SLA) and dihydroxyacetone phosphate (DHAP) by an SFP aldolase YihT. Finally, the SLA is reduced via an NADH-dependent SLA reductase (YihU) to DHPS, which is excreted from microorganisms. These four genes *YihSVTU* were annotated through K18479, K18478, K01671, and K08318 Orthology in KEGG, respectively.

#### Sulfo-ED Pathway

This pathway starts with an NAD^+^-dependent SQ dehydrogenase (EC:1.1.1.390) oxidizing SQ to 6-sulfogluconolactone (SGL). The SGL is hydrolyzed to 6-deoxy-6-sulfogluconate (SG) by an SGL lactonase (EC:3.1.1.99). The SG is then converted by an SG dehydratase (EC:4.2.1.162) to 2-keto-3,6-deoxy-6-sulfo-gluconate (KDSG). The KDSG is cleaved by a KDSG aldolase (EC:4.1.2.58) into pyruvate and 3-SLA. The SLA can be oxidized by a NAD^+^-dependent SLA dehydrogenase (EC:1.2.1.97) to SL. The reference sequences of these enzymes were collected manually from Uniprot database^5^.

#### SFT Pathway

Three key enzymes take part in this pathway. The SQ is converted to SF by an aldose/ketose isomerase, which is the same enzyme as the first step of sulfo-EMP pathway. SF is cleaved to 3-SLA by SF transaldolase enzyme. Finally, The SLA is oxidized by a NAD^+^-dependent SLA dehydrogenase to SL. The SLA dehydrogenase is the same enzyme as the last step of sulfo-ED pathway. The reference sequence of SF transaldolase enzyme was collected from IMG^6^ according to Frommeyer et al. (2020).

Isoprene (2-methyl-1, 3-butadiene) is an important volatile organic compound emitted to the atmosphere, and has significant effect on climate (Carrión et al., 2018). Isoprene may be degraded by microbial communities with the isoprene monooxygenase (IsoMO). The gene *isoA* encoding the α-subunit of IsoMO was selected as a marker gene for distribution, diversity, and abundance of the isoprene-degrading pathway in the environment (Carrión et al., 2018, 2020). The reference sequences of IsoA enzyme were collected manually from NCBI according to Carrión et al. (2018).

### The Processing of Simulated Benchmark and Natural Real Datasets

To verify the accuracy of DiTing in evaluating the relative abundance of biogeochemical pathways, CAMISIM (Fritz et al., 2019) was used to simulate five metagenomic shotgun sequenced samples using 15 genomes. These 15 genomes can be divided into three groups (photoautotrophs, chemoautotrophs, and heterotrophs). The photoautotrophic group was made up of five *Cyanobacteria* genomes (NCBI accession numbers: GCF_000018105.1, GCF_000020025.1, GCF_000021825.1, GCF_000317105.1, and GCF_000317615.1). The chemoautotrophic group was made up of five ammonia-oxidizing archaea (AOA) genomes (NCBI accession numbers: GCF_000299365.1, GCF_000299395.1, GCF_000875775.1, GCF_000956175.1, and GCF_013407185.1). The heterotrophic group was made up of five SAR11 genomes (NCBI accession numbers: GCF_000012345.1, GCF_000195085.1, GCF_000299095.1, GCF_000299115.1, and GCF_012276695.1). The metagenomic samples were simulated according to the relative abundance ratio of *Cyanobacteria*:SAR11:AOA genomes. Finally, CAMISIM created Illumina 2 × 150 bp paired-end reads with a size of 2 Gb for each simulated sample. These five simulated metagenomic samples were then fed into DiTing (default parameters) to produce the relative abundance of KO families and pathways. Due to the lack of features to specify the relative abundance of genes or pathways in CAMISIM, we manually inferred KO relative abundance profiles as the real result. To this end, all the 15 genomes were annotated by KofamScan software (Aramaki et al., 2019) to infer the KO family. The KO relative abundance profile from each simulated sample can be inferred according to the KofamScan annotation and relative abundance of genomes used in the simulation. KO relative abundance profile similarity between the DiTing output and the real result was calculated with Pearson’s correlation coefficient (PCC).

Subsequently, we applied DiTing to the natural real metagenomic datasets from the hydrothermal vent and *Tara* Ocean project. The raw reads were first filtered and trimmed by Trimmomatic v3.6 (Bolger et al., 2014). The clean reads were then fed into DiTing using default parameters: diting.py –r<clean reads dir> -o<diting.out dir>, where <clean reads dir> is the directory containing a set of clean reads files, <diting.out dir> is the directory for output., The clean reads were assembled using Megahit v1.1.2 (Li et al., 2016) under default parameters in DiTing.

We also tested DiTing on metatranscriptomic datasets. Three published and analyzed metagenomes with their corresponding metatranscriptomes were selected. These data were derived from hydrothermal vent fluid samples at Axial Seamount located on the Juan de Fuca Ridge in the Pacific Northwest region (Fortunato et al., 2018). They were selected as the data have been analyzed with a comprehensive functional prediction regarding biogeochemical cycles, thus facilitating comparison with the results generated by DiTing. First, the metagenomic reads were assembled using Megahit v1.1.2 with default parameters (Li et al., 2016). Second, the metagenomic contigs and the corresponding metatranscriptomes were used as input to DiTing. Then DiTing was run by: diting.py –r <metatranscriptomic clean reads dir> -a<metagenomics assembled contigs dir> -o<diting.out dir>, where <metatranscriptomic clean reads dir> is the directory containing the three metatranscriptomic clean reads files, <metagenomics assembled contigs dir> is the directory containing the three metagenomic assembled contigs files, and <diting.out dir> is the directory for output.

## Results and Discussion

### General Information of DiTing

We developed a new metagenomics/metatranscriptomic analysis pipeline, DiTing, to infer and compare the prevalence of genes and pathways of key biogeochemical cycles. DiTing consists of four main features: (i) automated assembly, Open Reading Frame (ORF) prediction, mapping, and gene annotation from reads; (ii) a manually created and curated DMSP cycling-related gene database; (iii) the specific formulae for DMSP and other biogeochemical pathways to calculate the relative abundance of biogeochemically relevant pathways and genes; and (iv) visualization of results comparing biogeochemical cycling potential between different inputted samples. These features make DiTing a flexible and versatile tool wrapper for studying biogeochemical cycles, or just as a platform to tackle metagenomic shotgun sequencing data. Additionally, DiTing has high speed. Five samples (from the hydrothermal vent case study below) that are ∼500 Gb in total were used to evaluate the speed. The total run time for all analyses from reads to visualization was ∼33 h using 60 CPU threads on a Linux version 4.15.0-20-generic server (Ubuntu 18.04; CPU, Intel(R) Xeon(R) Gold 6140 CPU @ 2.30GHz; RAM, 256 GB).

### Accuracy Testing of DiTing Using Simulated Benchmark Datasets

**FIGURE 2:**
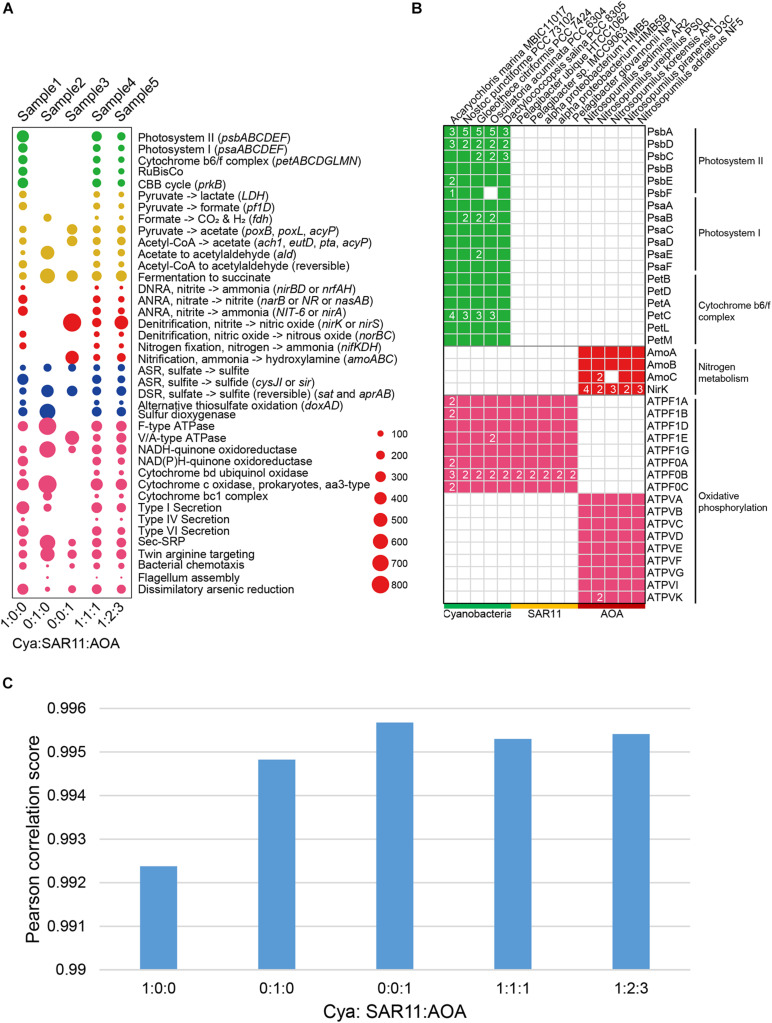
**(A)** Bubble plots depicting the DiTing result of the relative abundance of pathways in simulated metagenomes. The horizontal axis indicates the relative abundance ratio of *Cyanobacteria*:SAR11:AOA genomes used in simulated metagenomes. Cya, *Cyanobacteria*; SAR11; *Pelagibacterales*; AOA. ammonia oxidation archaea; DNRA, dissimilatory nitrate reduction to ammonium; ANRA, assimilatory nitrate reduction to ammonium; ASR; assimilatory sulfate reduction; DSR; dissimilatory sulfate reduction. **(B)** Selected genes distributed among 15 genomes used to simulate metagenomes. 15 genomes were divided into three groups (*Cyanobacteria*, SAR11 and AOA). The genes were annotated by KofamScan. **(C)** Pearson correlation between gene relative abundance outputted from DiTing and that predicted through the relative abundance of genomes for simulation manually.

To verify the accuracy of DiTing, we compared DiTing’s result on the simulated data with genetic features of 15 genomes and manually inferred the KO family relative abundance profiles (Figure 2). The overall relative abundances of biogeochemical pathways in simulated samples were consistent with the genetic features of genomes used in the simulation (Figures 2A,B). For example, metagenomes in sample 1, 4, and 5 possessed photosynthesis-related pathways (photosystem I, II, and cytochrome *b_6_/f* complex), which were absent in sample 2 and 3 (Figure 2A). This is because genomes used to simulate sample 1, 3, and 5 contained *Cyanobacteria*, which is a photoautotrophic organism possessing photosynthesis-related genes (Figure 2B). Since only *Cyanobacteria* genomes were used to simulate sample1 metagenome, the relative abundance of photosynthesis-related pathways in sample1 was highest (Figure 2A). Similarly, sample3 was simulated by only AOA, a typical bacterial ammonia oxidizer that possesses *amoABC* genes encoding the ammonia monooxygenase complex (Figure 2B). Correspondingly, the relative abundance of ammonia oxidation pathway was highest in sample 3, while it was absent in sample 1 and 2 that do not contain AOA (Figure 2A). The *nirK* gene encoding nitrite reductase was found with multiple copies in AOA genomes (Figure 2B). Consistently, this gene also showed a very high relative abundance in sample 3, in which the metagenome was simulated only by AOA genomes. Additionally, bacteria and archaea normally use F-type ATPase and V/A-type ATPases (Figure 2B) to hydrolyze ATP to ADP, respectively (Pisa et al., 2007; Fillingame, 1997). As expected, F-type ATPase was detected in samples simulated by genomes containing *Cyanobacteria* and SAR11 genomes (sample 1, 2, 4, and 5), and V/A-type ATPase was detected in samples simulated by genomes containing AOA genomes (sample 3, 4, and 5).

Subsequently, the translated gene sequences (amino acid) from 15 genomes for simulation were annotated using KofamScan software (Aramaki et al., 2019). Considering that CAMISIM (Fritz et al., 2019) lacks the feature to specify the relative abundance of genes or pathways directly, and there are no other appropriate tools available to achieve this to the best of our knowledge. We manually inferred the relative abundance of the KO family in simulated metagenomes according to KofamScan annotation and relative abundance of genomes, as the real KO relative abundance (Supplementary Table 2). On the other hand, we fed these five simulated metagenomic samples into DiTing to generate KO relative abundance profile. For a comparison of KO relative abundance profile produced by DiTing with the real one, the similarity between these two KO relative abundance profiles was calculated with PCC. All the PCC scores were higher than 0.99 (Figure 2C), which indicated the KO relative abundance profiles created by DiTing were strongly consistent with the real result. The above results verify the accuracy of DiTing.

### Application of DiTing on Five Real Hydrothermal Vent Datasets

**FIGURE 3:**
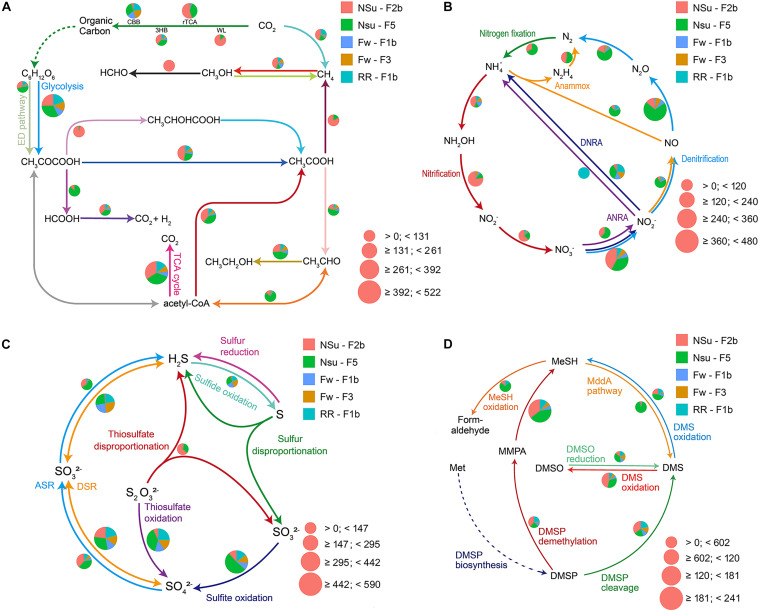
Pie charts representing the relative abundance of carbon **(A)**, nitrogen **(B)**, sulfur **(C)**, and DMSP **(D)** cycle-related pathways for five metagenomic samples from the Manus Basin. Normalized relative abundance was calculated through dividing the relative abundance of a pathway in an individual sample by the sum of this pathway’s relative abundance in all samples. The pie chart area reflects the relative abundance of the process according to the scale shown in pink. The dashed line in panel **(D)** means the data was not shown. **(A)** CBB, Calvin-Benson-Bassham cycle; rTCA, reductive citric acid cycle; WL, Wood-Ljungdahl pathway; 3HB, 3-hydroxypropionate bicycle. **(B)** ANRA, assimilatory nitrate reduction to ammonia; DNRA, Dissimilatory nitrate reduction to ammonia; Anammox, anaerobic ammonia oxidation. **(C)** ASR, assimilatory sulfate reduction; DSR, dissimilatory sulfate reduction. **(D)** DMSP, dimethylsulfoniopropionate; MMPA, methylmecaptopropionate; MeSH, methanethiol; DMSO, dimethyl sulfoxide; *L*-Met, *L*-methionine. This figure was the output from DiTing.

DiTing was used to analyze the biogeochemical potential of five marine metagenomic samples (Supplementary Table 3; NCBI accession number: ERR1679394-1679398) generated from hydrothermal vent samples taken at PACManus and North Su fields in the Manus Basin (Meier et al., 2017). The metagenomic clean reads ranged in size from 81 to 112 Gbp from each sample. The reads were assembled into 799,269 to 1,182,847 contigs with the total assembly sizes ranging from 0.58 to 1.00 Gbp. A total of 5,639,558 ORFs within these contigs were then predicted. ∼18.9% (1,065,097) ORFs were annotated against KEGG databases and affiliated to 8128 KO entries. The relative abundances of ∼100 biogeochemically relevant pathways were calculated (Supplementary Table 4) according to our new formulae (Supplementary Table 1). The relative abundance of genes within these pathways was also prepared for further analyses at the gene level (Supplementary Table 5). The summary sketch for visualization of these pathways was generated by DiTing (Figure 3), and these reflected the different patterns of community function within metagenomic samples.

**FIGURE 4:**
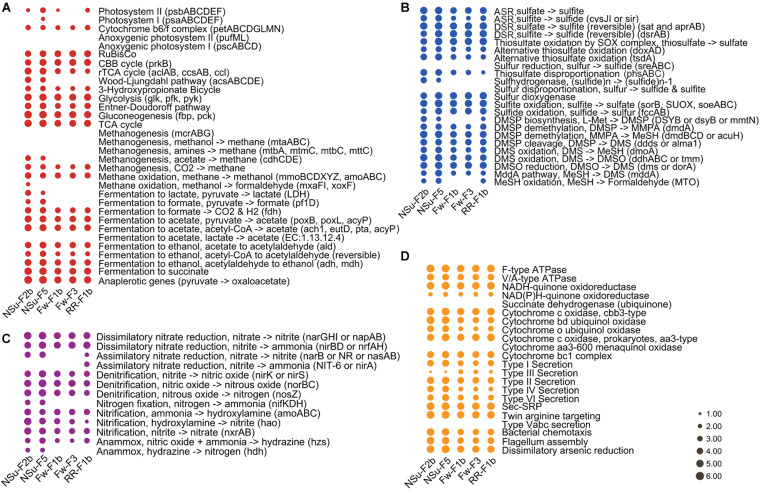
Bubble plots depicting the relative abundance of pathways for carbon **(A)**, sulfur **(B)**, nitrogen **(C)**, and other selected **(D)** processes. The key marker genes used to report on the genetic potential for pathways (as the relative abundances) are indicated in brackets. ASR, assimilatory sulfate reduction; DSR, dissimilatory sulfate reduction. The full name of these key marker genes can be found in Supplementary Table 1. For better visualization, we multiply the relative abundance by 10^3^ and transformed it by log(10).

Of the five metagenomes collected in diffuse hydrothermal vent fluids, NSu-F2b and NSu-F5 originated from acidic samples with sulfide (1.6 and 0.7 mmol l^–1^ H_2_S, respectively) and methane (0.2 and 0.01 mmol l^–1^ CH_4_, respectively) levels detected (Supplementary Table 3). The Fw-F1b, Fw-F3, and RR-F1b metagenomes originated from sites with no detectable H_2_S and CH_4_. Reassuringly, the NSu-F2b and NSu-F5 samples, with similar environmental parameters, showed the most similar distribution patterns for genes and pathways involved in the cycling of nitrogen, carbon, and sulfur (Figures 3, 4). Indeed, hierarchical clustering of samples according to their microbial function composition showed NSu-F2b and NSu-F5 fall into one cluster, and the other three samples into another cluster (Supplementary Figure 1).

At hydrothermal vents, chemolithoautotrophic microorganisms carry out carbon fixation coupled with oxidation of reduced sulfur compounds (Meier et al., 2017). In accordance, we found the relative abundance of thiosulfate oxidation, sulfite oxidation, and the first step of dissimilatory sulfate reduction pathways (reversible conversion of sulfate to sulfite) to be more highly represented compared to other sulfur cycle pathways in all five samples (Figures 3, 4). This indicated that sulfate reduction and sulfur oxidation were major processes in microbial sulfur cycling. This finding is supported by the presence of sulfate-reducing *Nitrospira* and sulfur-oxidizing *Gammaproteobacteria* dominating microbial communities at these hydrothermal vents (described in Meier et al., 2017, 2019). In addition, assimilatory sulfate reduction and thiosulfate disproportionation pathways were found only in NSu-F2b and NSu-F5 (Figure 3); the only samples with detectable sulfide levels, indicating microbes in these samples may incorporate sulfide into the amino acids cysteine (Cys) or homo-Cys. Here, the relative abundance of thiosulfate disproportionation was estimated by dividing the sum of relative abundance of *phsABC* by the number (*n* = 3) of essential subunits. The relative abundances of each subunit of thiosulfate reductase were often not equal to each other in the metagenomes (Supplementary Table 5). For example, *phsA* (encoding thiosulfate reductase subunit A) was always far more abundant than *phsC* (thiosulfate reductase cytochrome B subunit), and *phsB* (thiosulfate reductase electron transport protein) was not detected in any sample. This may be due to insufficient sequencing depth and/or protein redundancy. Whatever the reason for these discrepancies, it cannot be easily solved by bioinformatics alone and culture-dependent work is necessary. This phenomenon highlighted for the thiosulfate disproportionation genes may occur also in other pathways; thus further analyses at the gene level, not only at the pathway level, are essential in predicting the biogeochemical potential of microbial communities after DiTing analysis.

In previously tested seawater and sediment samples, known DMSP synthesis genes were always much less abundant than those for its catabolism (Curson et al., 2017, 2018; Williams et al., 2019). This was not the case in previously studied hydrothermal samples (Song et al., 2020), with the DMSP lyase gene *dddP* being the only detected DMSP catabolic gene. In three out of five hydrothermal samples interrogated here, the genetic potential to synthesize DMSP, through prokaryotic *dsyB* and *mmtN* genes, is far less than that for DMSP catabolism (DMSP synthesis:DMSP catabolism = 1:16.9) and not so dissimilar to ratios seen in seawater samples (Curson et al., 2017, 2018; Williams et al., 2019). The reasons for this discrepancy between the distinct samples are unknown. The prokaryotic DsyB sequences retrieved from these data were clustered with ratified DsyB proteins, not with eukaryotic DSYB and non-functional DsyB-like proteins from *Streptomyces varsoviensis*, which support their function in DMSP synthesis (Supplementary Figure 2). Interestingly, sample NSu-F2b has higher DMSP synthesis potential than any other samples due to relatively high levels of bacteria with *mmtN*. As discussed by Song et al. (2020), the potential for DMSP cleavage was more prominent than for DMSP demethylation (*dmdA*) in all hydrothermal samples, although catabolism of MMPA, the initial product of DMSP demethylation by DmdA (Howard et al., 2006), was very abundant. These data support DMSP cleavage being the dominant DMSP catabolic pathway in hydrothermal sediments, as proposed in Song et al. (2020). Alternatively, there could be novel DMSP demethylase enzymes. This would explain why there were such low *dmdA* levels in hydrothermal sediment, yet very high MMPA degradation potential. The potential for oxidation and reduction of DMSP catabolites, DMS and methanethiol was similar to that described in Song et al. (2020), with sites NSU-F2b and F5 showing the greatest potential. Thus, some interesting predictions of DMSP cycling were enabled by DiTing analysis on the metagenomes analyzed here. It should be emphasized that any predictions made from genetic potential alone require further investigation regarding function and expression and, importantly, substantiation for synthesis and turnover rate analysis.

The samples NSu-F2b and NSu-F5 had lower oxygen concentration than Fw-F1b, Fw-F3, and RR-F1b samples, especially NSu-F2b (0.07 and 0.14 mmol l^–1^ for NSu-F2b and NSu-F5, respectively; 0.17–0.2 mmol l^–1^ for other three). Indeed, compared to the other three samples, NSu-F2b and NSu-F5 had significantly more genes encoding *bd* ubiquinol cytochrome oxidases (*p* < 0.01) that are associated with low oxygen concentrations (Figure 4). It is worth noting that the *bd* oxidase was enriched most in NSu-F2b under the highest sulfide concentration (1.6 mmol l^–1^) and lowest oxygen concentration. A previous study found that *bd* oxidase could promote sulfide-resistant O_2_ consumption and growth in *Escherichia coli* (Forte et al., 2016) implying the important role of *bd* oxidases in the low oxygen NSu-F2b environment.

The NSu-F2b and NSu-F5 samples showed enrichment for denitrification, nitrification, and nitrogen fixation potential, which may be due to the lower oxygen levels of these samples or is possibly reflecting the nitrogen availability at higher temperatures. Notably in NSu-F5, genes encoding for the denitrification enzymes responsible for the reduction of the cytotoxic gaseous intermediates, nitric oxide (NO), *norBC*, and nitrous oxide (N_2_O), *nosZ*, are significantly enriched. These are alongside the nitrifying genes responsible for aerobic conversion of nitrite to nitrate (*nxrAB*). Genes encoding the nitrification enzymes involved in ammonia oxidation process, *amoABC*, hydroxylamine, *hao*, nitrate, *nxrAB*, are significantly enriched in The NSu-F2b and NSu-F5 samples. The importance of denitrification and nitrification to the nitrogen cycling in hydrothermal vents has previously been reported (Bourbonnais et al., 2012). These metagenomes highlight the metabolic importance of nitrogen cycling with the potential for all other pathways being at similarly high levels (Supplementary Table 4) in all samples with the exception that nitrite assimilation (nitrite to ammonia) was not detected. Again, this may reflect nitrogen availability but is also indicative of nitrogen source preference of the microbiomes under the highly reactive physicochemical constraints of the vent environment. This study illustrates the need for comprehensive measurements of nitrogen flux, metatranscriptomic analyses to ascertain the most active pathways, and identification of the dominant organisms responsible for nitrogen cycling in these ecosystems. Overall, these results highlight potential microbial metabolic differences in communities from different hydrothermal samples that most likely reflect changes in environmental conditions.

### Application of DiTing on 15 Real *Tara* Ocean Project Datasets

DiTing was also applied to analyze 15 metagenomic samples from chlorophyll *a* (*Chla*) maximum layer in Mediterranean Sea from *Tara* Ocean project. The metagenomic clean reads ranged in size from 1.24 to 52.53 Gbp from each sample. The reads were assembled into 71,183–1,601,956 contigs with the total assembly sizes ranging from 0.045 to 1.38 Gbp. A total of 18,431,131 ORFs within these contigs were then predicted. ∼24% (1,065,097) ORFs were annotated against KEGG databases and affiliated to 8759 KO entries. The 74 pathways related to biogeochemical cycles were found (Supplementary Table 6). Compared to the sample-derived hydrothermal vents, the *Chla* maximum layer contains a remarkably high relative abundance of photosystem pathways as expected (Supplementary Tables 6, 7). Additionally, the eukaryotic DMSP synthesis gene, *DSYB* was detected in 10 out of 15 *Chla* maximum samples, which were absent in the hydrothermal vent samples. The relative abundance of *DSYB* was comparable to that of prokaryotic DMSP synthesis gene *dsyB* in *Chla* maximum layers (Supplementary Table 7), indicating that the DMSP was produced by both prokaryotes and eukaryotes in these environments. For DMSP degradation, in six out of 15 samples, the genetic potential to demethylate DMSP, through the *dmdA* gene, was higher than that for DMSP cleavage (*ddds* and *alma1*) (DMSP demethylation:DMSP cleavage = 1.69:1). This contrasts with the hydrothermal vent samples. In another nine samples, the potential for DMSP demethylation was comparable to that for DMSP cleavage (DMSP demethylation:DMSP cleavage = 0.82:1). These data support both DMSP demethylation and cleavage being the dominant DMSP catabolic pathways in the *Chla* maximum layer.

### Application of DiTing on the Combination of Metagenomic and Metatranscriptomic Datasets

**FIGURE 5:**
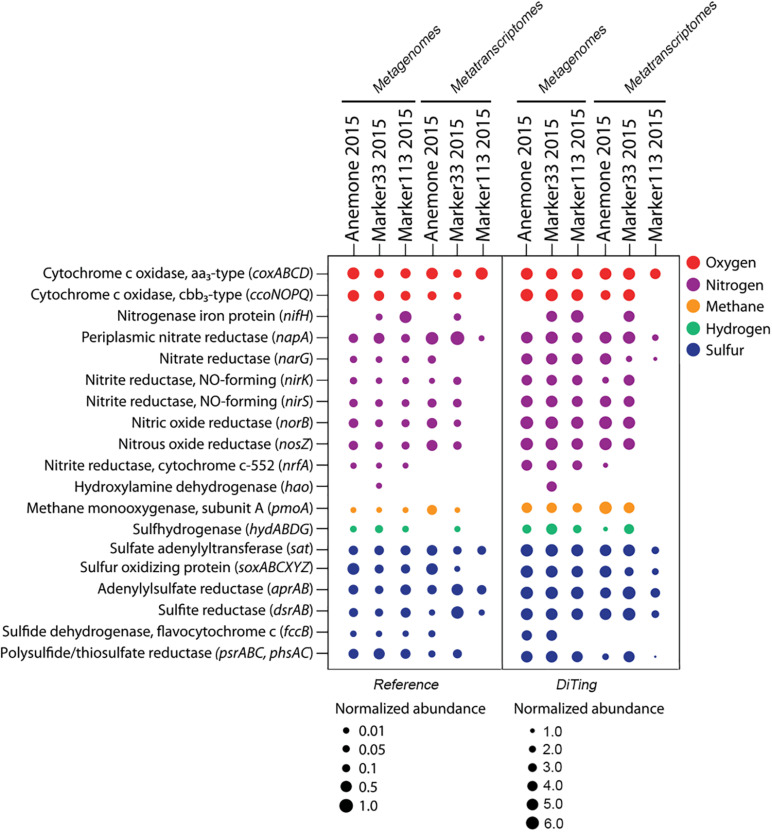
Comparative analysis of the combination of metagenomic and metatranscriptomic datasets between a reference study and DiTing. The left panel is taken from reference (Fortunato et al., 2018), and shows normalized abundance and transcription of key genes for oxygen, nitrogen, methane, hydrogen and sulfur metabolism in hydrothermal vent metagenomes and metatranscriptomes. The right panel shows the results produced using analysis by DiTing. The normalized method is different from the reference. Multiply the relative abundance by 10^3^ and transformed by log(10).

Subsequently, we applied DiTing on three metagenomic samples with their corresponding metatranscriptomes. The metagenomic and metatranscriptomic clean reads ranged in size from 6.8 to 9.9 Gb and 2.7 to 3.9 Gb for each sample, respectively. The total run time for all analyses from assembly to visualization was ∼11 h using 60 CPU threads on a Linux version 4.15.0-20-generic server (Ubuntu 18.04; CPU, Intel(R) Xeon(R) Gold 6140 CPU @ 2.30GHz; RAM, 256 GB). The overall relative abundance of biogeochemical pathways, after analysis with DiTing, was consistent with the original study (Figure 5; Fortunato et al., 2018). For example, the *cbb*_3_-type cytochrome *c* oxidase genes/transcripts were found in three samples but were absent in the Marker113 2015 metatranscriptomic samples according to both the DiTing output and the reference result. The gene for the nitrogenase iron protein (*nifH*) was absent in one metagenomic and two metatranscriptomic samples in both analyses. However, there were also some differences between the reference study and the DiTing results. For example, the nitrate reductase gene (*narG*) was absent in two samples according to Fortunato et al. (2018), but DiTing found it in all samples (Figure 5). Notably, the *narG* gene was present at extremely low levels in the Marker33 2015 and Marker133 2015 metatranscriptome samples, but phylogenetic analysis confirmed that their products cluster with ratified functional sequences instead of those without nitrate reductase activity (Supplementary Figure 3). These *narG* transcripts may have been missed in the original study due to the universal threshold (e-score of 1E-10, 30% amino acid identity and alignment length of 40 amino acids) used for all gene annotation against the KO database. In contrast, DiTing employed the specific cutoff threshold (—domT 304.50) for *narG* according to Kofam suggestion (Aramaki et al., 2019), enabling the correct annotation.

## Conclusion

In summary, this study developed a pipeline (DiTing) to infer and compare biogeochemical pathways from metagenomic and metatranscriptomic data. DiTing is a portable tool for analyzing metagenomic and metatranscriptomic datasets, providing automatic, multi-threaded bioinformatic workflows for data handling, including read assembly, ORF prediction, annotation, and customized specific formulas for calculating the relative abundance of biogeochemical pathways. The visualization module is designed to more easily compare functions between samples via graphical outputs. Additionally, a verified database was built manually for the annotation of genes involved in the production and cycling of DMSP. As validation of the outputs produced by DiTing, comparisons of the relative abundance of biogeochemical pathways in published metagenomes to those calculated by DiTing were consistent. By applying DiTing to analyze five hydrothermal shotgun metagenomes, we showed that the functional profile could accurately reflect changes in environmental conditions (H_2_S and O_2_ concentrations). Besides marine environments, DiTing was supposed to be applied easily to other interesting environments (e.g., glaciers, soil environments, and wastewater). DiTing can be applied readily to metagenomic and/or metatranscriptomic studies with relatively straightforward user intervention. This bioinformatics framework will facilitate our understanding of spatial and temporal changes in microbiome-mediated biogeochemical cycles.

## Data Availability Statement

Sequence data for genomes used to simulate benchmark datasets can be found in NCBI under accession numbers: GCF_000018105.1, GCF_000020025.1, GCF_000021825.1, GCF_ 000317105.1, GCF_000317615.1, GCF_000299365.1, GCF_000 299395.1, GCF_000875775.1, GCF_000956175.1, GCF_013 407185.1, GCF_000012345.1, GCF_000195085.1, GCF_00029 9095.1, GCF_000299115.1, and GCF_012276695.1. Sequences data for metagenome of five hydrothermal vent datasets can be found in NCBI Sequence Read Archive under accession numbers ERR1679394-1679398. Sequences data for metagenome of 15 Tara Ocean project datasets can be found in NCBI Sequence Read Archive under accession numbers ERR315856, ERR315859, ERR315860, ERR318618, ERR318619, ERR318620, ERR318621, ERR594315, ERR594329, ERR598950, ERR599073, ERR599092, ERR599094, ERR599095, and ERR599153. Additionally, the DiTing software has been deposited in https://github.com/xuechunxu/DiTing.

## Author Contributions

X-HZ conceived the project and designed the study. C-XX and HL implemented the software and drafted the manuscript. X-YZ, JL, YZ, GR, JT, and ML provided great help on data presentation and manuscript writing. All authors read and approved the final manuscript.

## Conflict of Interest

The authors declare that the research was conducted in the absence of any commercial or financial relationships that could be construed as a potential conflict of interest.

## Publisher’s Note

All claims expressed in this article are solely those of the authors and do not necessarily represent those of their affiliated organizations, or those of the publisher, the editors and the reviewers. Any product that may be evaluated in this article, or claim that may be made by its manufacturer, is not guaranteed or endorsed by the publisher.

## Footnotes

1
https://github.com/xuechunxu/DiTing

2
https://docs.conda.io

3
ftp://ftp.genome.jp/pub/db/kofam/

4
https://github.com/xuechunxu/DiTing/blob/master/Pathway_formulas.txt

5
https://www.uniprot.org/

6
https://img.jgi.doe.gov/
