# Supplementary material for: DiTing: A Pipeline to Infer and Compare Biogeochemical Pathways From Metagenomic and Metatranscriptomic Data
Source: Front Microbiol. 2021 Aug 2;12:698286. doi: 10.3389/fmicb.2021.698286 (PMC8367434; doi:10.3389/fmicb.2021.698286)
Supplement: Supplementary file 3 [file Table_3.DOCX]

**Table S3:** A summary of sampling sites and environmental parameters for collected samples (Meier et al., 2017)

| **Sample name** | **Sample type** | **Latitude** | **Longitude** | **Depth [m]** | **T [℃]** | **pH** | **H_2_S [mM]** | **CH_4_ [mM]** | **DIC [mM]** | **O_2_ [mM]** |
| --- | --- | --- | --- | --- | --- | --- | --- | --- | --- | --- |
| NSu-F2b | water/fluid | S 03°47.995' | E 152°06.052' | 1155 | 51.7 | 4.3 | 1.61 | 0.2 | 3.07 | 0.07 |
| NSu-F5 | water/fluid | S 03°47.955' | E 152°06.080' | 1199 | 31.4 | 5.1 | 0.7 | 0.01 | 0.18 | 0.14 |
| Fw-F1b | water/fluid | S 03°43.700' | E 151°40.344' | 1709 | 3.7 | 6.5 | 0 | 0 | 0.24 | 0.17 |
| Fw-F3 | water/fluid | S 03°43.698' | E 151°40.350' | 1705 | 3.2 | 7.2 | ND | ND | ND | ND |
| RR-F1b | water/fluid | S 03°43.238' | E 151°40.519' | 1685 | 6.6 | 7.5 | 0 | 0 | 2.34 | 0.2 |

ND – ‘not determined’. 0 – below detection limit

Meier, D. V., Pjevac, P., Bach, W., Hourdez, S., Girguis, P. R., Vidoudez, C. et al. (2017). Niche partitioning of diverse sulfur-oxidizing bacteria at hydrothermal vents. *ISME J.* 11, 1545-1558. doi: 10.1038/ismej.2017.37
