# Supplementary material for: DiTing: A Pipeline to Infer and Compare Biogeochemical Pathways From Metagenomic and Metatranscriptomic Data
Source: Front Microbiol. 2021 Aug 2;12:698286. doi: 10.3389/fmicb.2021.698286 (PMC8367434; doi:10.3389/fmicb.2021.698286)
Supplement: Supplementary file 8 [file Table_8.DOCX]

**Supplementary materials**


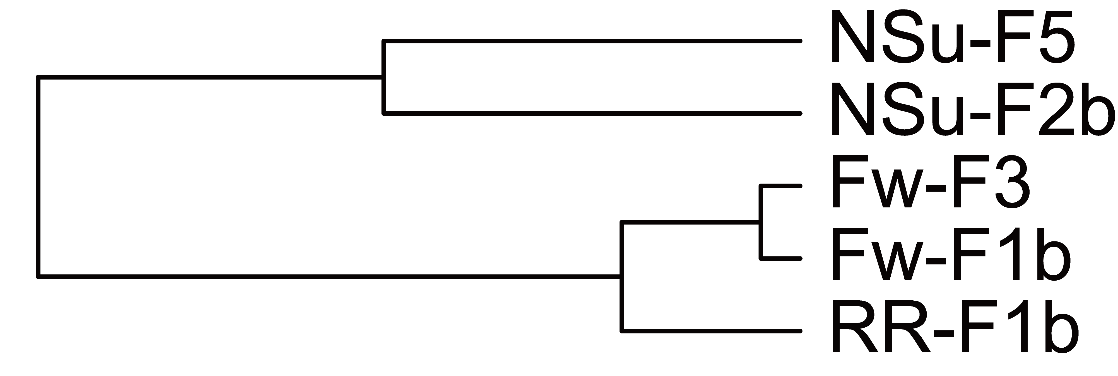


**Fig. S1.** Cluster dendrogram depicting the average linkage hierarchical clustering based on a Bray-Curtis dissimilarity matrix of the relative abundance of ~ 100 biogeochemical pathways produced by DiTing.


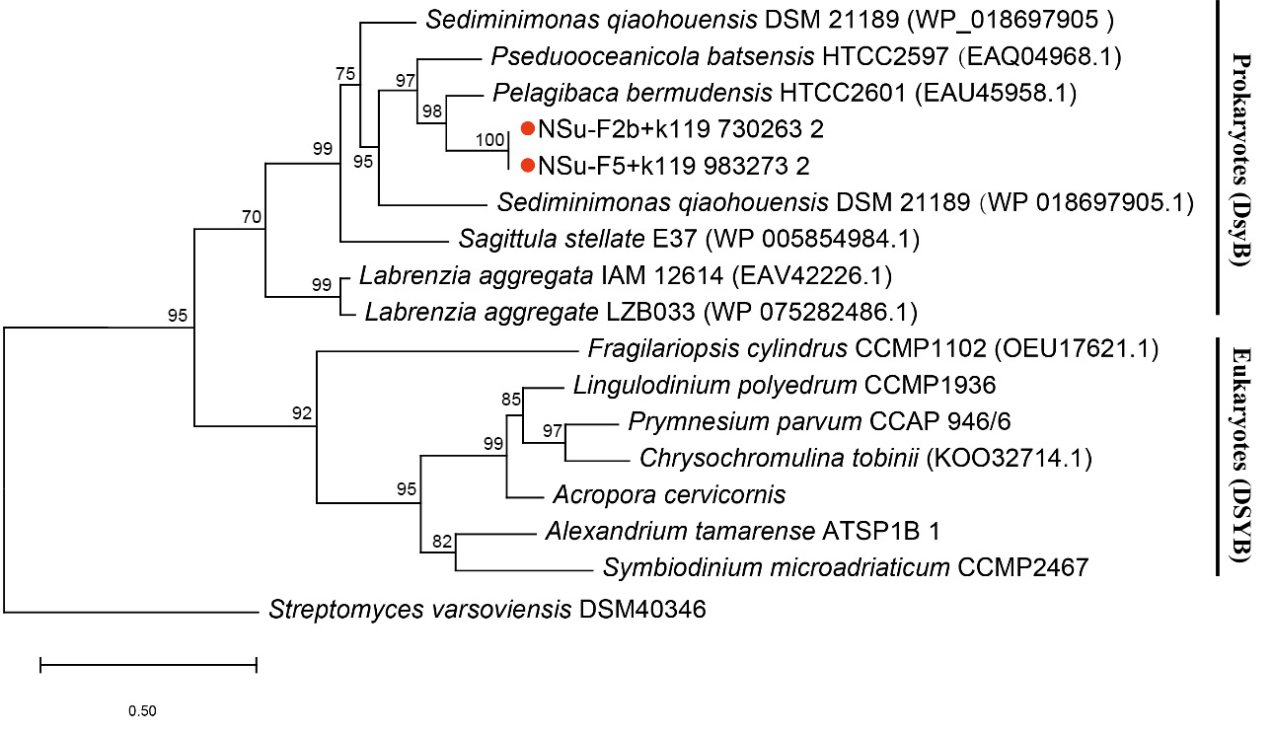


**Fig. S2.** Maximum likelihood phylogenetic tree of algal DSYB and bacterial DsyB proteins, built by FastTree software. Ratified proteins of homologous DsyB (in bacteria) and DSYB (in algae) genes available from NCBI alongside the newly retrieved sequences from metagenomes were used for phylogenetic tree reconstruction. DSYB The proteins derived in this study are marked with a red solid circle.


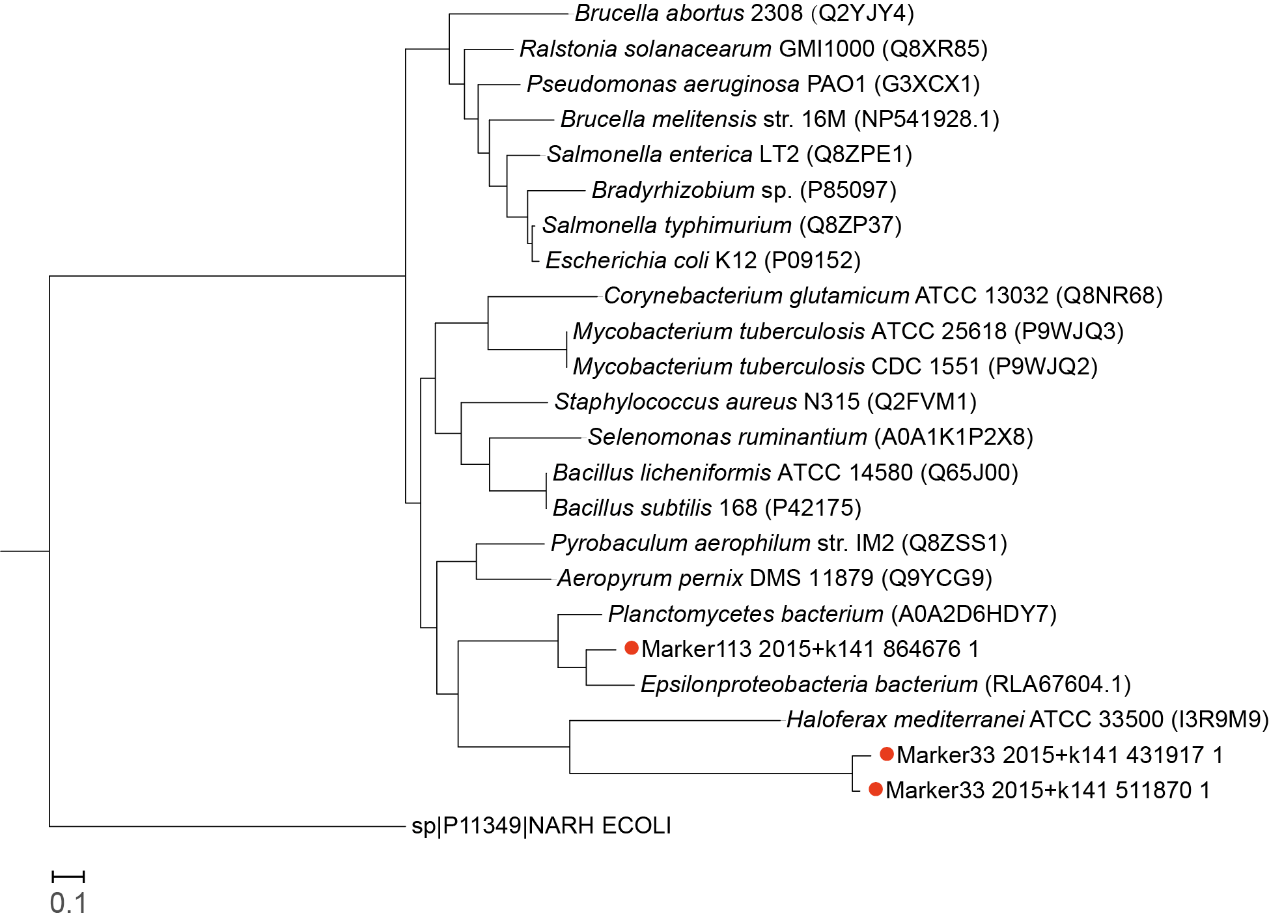


**Fig. S3.** Maximum likelihood phylogenetic tree of NarG. Ratified proteins of NarG available from Uniprot alongside the newly retrieved sequences from corresponding metagenomes for metatranscriptomes were used for phylogenetic tree reconstruction. The proteins derived in this study are marked with a red solid circle.

**Table S1** The formula for each pathway. A detailed explanation can be found at the website (<https://github.com/xuechunxu/DiTing/blob/master/Pathway_formulas.txt>). The KEGG Orthology and Gene names in formula are their relative abundance.

**Table S2** The functional comparison of the simulated data produced by DiTing with the real result.

**Table S3:** A summary of sampling sites and environmental parameters for collected samples.

**Table S4** The relative abundance of biogeochemical pathways in metagenomes from hydrothermal vent fluids at PACManus and North Su fields in Manus Basin.

**Table S5** The relative abundance of genes within pathways analyzed for collected samples. Metagenome samples were collected from hydrothermal vent fluids at PACManus and North Su fields in Manus Basin.

**Table S6** The relative abundance of biogeochemical pathways in metagenomes from the *Tara* Ocean project.

**Table S7** The relative abundance of genes within pathways analyzed for collected samples. Metagenome samples were collected from deep chlorophyll maximum layer in Mediterranean Sea from *Tara* Ocean project.
